# Simulation and validation of spinal construct testing based on ASTM F1717

**DOI:** 10.3389/fbioe.2025.1673061

**Published:** 2025-11-26

**Authors:** Byeong Cheol Jeong, On Sim, Chiseung Lee

**Affiliations:** 1 Department of Biomedical Engineering, Graduate School, Pusan National University, Busan, Republic of Korea; 2 Department of Biomedical Engineering, School of Medicine, Pusan National University, Busan, Republic of Korea; 3 In Silico Medicine Lab, Biomedical Research Institute, Pusan National University Hospital, Busan, Republic of Korea

**Keywords:** ASTM F1717, spinal implant, static compression and tension, finite element analysis, computational modeling, simulation

## Abstract

**Introduction:**

Spinal fixation constructs must demonstrate reliable mechanical performance before clinical use. ASTM F1717 provides a standardized vertebrectomy model for comparative static evaluation. This study assessed construct mechanics and examined whether validated finite element analysis (FEA) can support simulation based design decisions.

**Methods:**

Constructs comprised Ti-6Al-4V ELI pedicle screws and rods; the support blocks were UHMWPE. Testing followed ASTM F1717 under static compression and tension at 25 mm/min. Four screw configurations varied diameter and length (640, 650, 740, 750). A model form investigation compared a jig included model (JIM) and a jig excluded model (JEM) to quantify the tradeoff between accuracy and efficiency.

**Results:**

Experiment–FEA agreement was strong across metrics, with errors in most cases below 5 percent; the largest deviation was 7.12 percent for the flexion force at 20 mm in the 740 group. Plastic deformation of the rod occurred before 20 mm, which supports using the reaction force at 20 mm as a robust comparator. Increasing screw diameter produced larger reaction forces than increasing screw length, while greater construct stiffness was associated with shorter yield displacement of the rod. Relative to JIM, JEM reduced computational time by about 20–32 percent while maintaining reaction force differences within about 0.3 percent in the verification runs.

**Discussion:**

Validated FEA reproduced ASTM F1717 construct behavior within prespecified acceptance bounds and can complement early design screening. The parametric analysis highlights diameter as the dominant geometric factor for construct level force response under the tested conditions, whereas length had a comparatively minor influence. These findings indicate that the evaluated screw configurations can enhance structural durability and surgical safety, and they support the use of validated FEA as a reliable alternative to selected physical tests during early stages of implant design.

## 1 Introduction

Low back pain is recognized as a major health issue that severely impairs quality of life and can lead to chronic disability (Eastell et al., 1991; Ballane et al., 2017). Although non-operative treatments can yield favorable clinical outcomes, surgical interventions generally offer superior long-term results when considering overall prognosis (Siebenga et al., 2006; Ni et al., 2010). Spinal implants play an essential role in restoring stability and functionality to the injured vertebrae during surgical treatment. These implants are primarily used to reinforce the spine after deformity correction, disc degeneration, trauma, or tumor resection, and are fabricated from biocompatible materials such as titanium alloys (Ti-6Al-4V), cobalt-chromium alloys (Co-Cr), polyetheretherketone (PEEK), and medical-grade silicone (Gaines., 2000).

Pedicle screw systems manufactured from these materials are indispensable components in spinal fixation surgeries, yet implant failures continue to be reported. Common failure mechanisms include rod fracture, screw loosening or breakage, and junctional problems; recent reports describe hardware failure rates ranging from 2.8% to 32%, increasing with procedure complexity (e.g., three-column osteotomies) (Mensah et al., 2024). Implant-associated infections remain a major postoperative complication. Accordingly, systematic evaluation and continuous improvement of the mechanical performance of spinal implants are imperative.

Mechanical testing using cadaveric specimens and clinical studies are traditionally employed to assess implant performance (Dienstknecht et al., 2011; Osterhoff et al., 2011; Saiki et al., 2002; Li et al., 2016). However, cadaveric testing has inherent limitations, including high biological variability among specimens and the inability to reuse samples due to unavoidable bone destruction during testing. To overcome these constraints, this study utilized ultra-high-molecular-weight polyethylene (UHMWPE) blocks to replace bone structures and performed static compression and tension tests in accordance with the ASTM F1717 standard. UHMWPE is recognized for its consistent mechanical properties across compression, bending, and fatigue tests, making it a widely accepted standard material for mechanical performance evaluations.

Consistent with the comparative intent of ASTM F1717, we mounted the construct on two UHMWPE support blocks, which are widely used because they are isotropic, easy to machine, and yield low data scatter. By contrast, ceramic-based surrogates, although they can approach cortical-bone stiffness and hardness, are brittle and difficult to machine and fixture, and may fail in a non-physiologic brittle manner in a vertebrectomy setup (Fu et al., 2013). Accordingly, UHMWPE blocks were used for all tests.

Despite its advantages, physical testing entails substantial material costs. Each test consumes four pedicle screws, two spinal rods, and two UHMWPE blocks, in addition to requiring expensive universal testing machines, thereby leading to high overall costs. Moreover, the time required for material procurement and experimental preparation significantly increases opportunity costs.

To address these limitations, this study proposes the application of computational modeling and simulation (CM&S) as an alternative to physical testing. Furthermore, this study aims to validate CM&S based on the ASTM F1717 standard as a viable alternative to physical testing. CM&S has been widely utilized in the research, development, and regulatory evaluation of medical devices, as well as in proposing optimal surgical strategies tailored to individual patients (Park et al., 2025; Kim et al., 2024; Jeong et al., 2023a; Sim et al., 2022; Jeong et al., 2023b; Lim et al., 2024; Day et al., 2024; Toriya et al., 2023; Morrison et al., 2018). In particular, finite element analysis (FEA) is a systematic tool for analyzing stress and deformation distributions within spinal implants, helping to mitigate issues related to inter-specimen variability and high experimental costs commonly encountered in physical testing. Furthermore, the ASTM F1717 standard provides a validated framework for mechanical performance evaluation, and when coupled with CM&S, it facilitates identification of worst-case conditions while reducing experimental testing burdens (ASTM International, 2020; ASTM International, 2016; ASTM International, 2019). In this context, this study conducts finite element simulations of spinal fixation constructs under the same static compression and tension conditions as in physical testing and compares the outcomes. Additionally, a series of parametric simulations varying pedicle screw diameter and length were conducted to identify optimal designs that minimize surrogate bone damage.

Several prior studies have advanced the credibility of spinal-implant modeling. Nagaraja et al. presented a component-level VVUQ exemplar for Ti-6Al-4V rods under three-point bending, establishing clear practices for verification, validation, and uncertainty quantification (Nagaraja et al., 2020). An ASME V&V 40 end-to-end example then linked model risk and credibility requirements to a pedicle screw system in compression–bending (Nagaraja et al., 2024). Foltz et al. combined experiments and FEA to examine posterior instrumentation and growing-rod constructs (Foltz et al., 2019). While these studies strengthen credibility for spinal-implant modeling, they do not collectively cover the specific questions addressed here. The rod-level VVUQ exemplar focuses on three-point bending rather than the full ASTM F1717 construct; the V&V 40 end-to-end example treats an F1717-relevant compression–bending context but does not examine a common modeling simplification such as jig omission; and none of the three provides an F1717-aligned, side-by-side parametric assessment of pedicle screw diameter and length with coordinated experiment–FEA comparison. To address these gaps, the present study operates within the ASTM F1717 configuration, quantifies agreement between experiments and FEA under static compression and tension, evaluates the impact of jig inclusion versus exclusion, and performs a targeted parametric study of screw diameter and length. By reporting construct-level metrics including stiffness, yield measures, and reaction force at 20 mm, we provide F1717-aligned evidence for early design screening and simulation-based decisions. The overall aim of this study is to validate CM&S based on the ASTM F1717 standard as an effective substitute for physical experiments and to contribute to the optimization of spinal fixation construct designs.

## 2 Methods

### 2.1 Mechanical testing

The spinal construct components used in this study, including pedicle screws, spinal rods, and tightening screws, were fabricated from Ti-6Al-4V ELI alloy (U.S. Titanium Industry Inc., 2022). Compared to standard Ti-6Al-4V, Ti-6Al-4V ELI offers superior ductility, impact resistance, and biocompatibility; while Ti-6Al-4V is primarily used for industrial applications, Ti-6Al-4V ELI is mainly utilized for medical implants and orthopedic devices (ASTM International, 2013; International Organization for Standardization, 2021). In addition, UHMWPE was employed as a surrogate for vertebral bone, and its material properties were verified through testing requested from the supplier.

**FIGURE 1:**
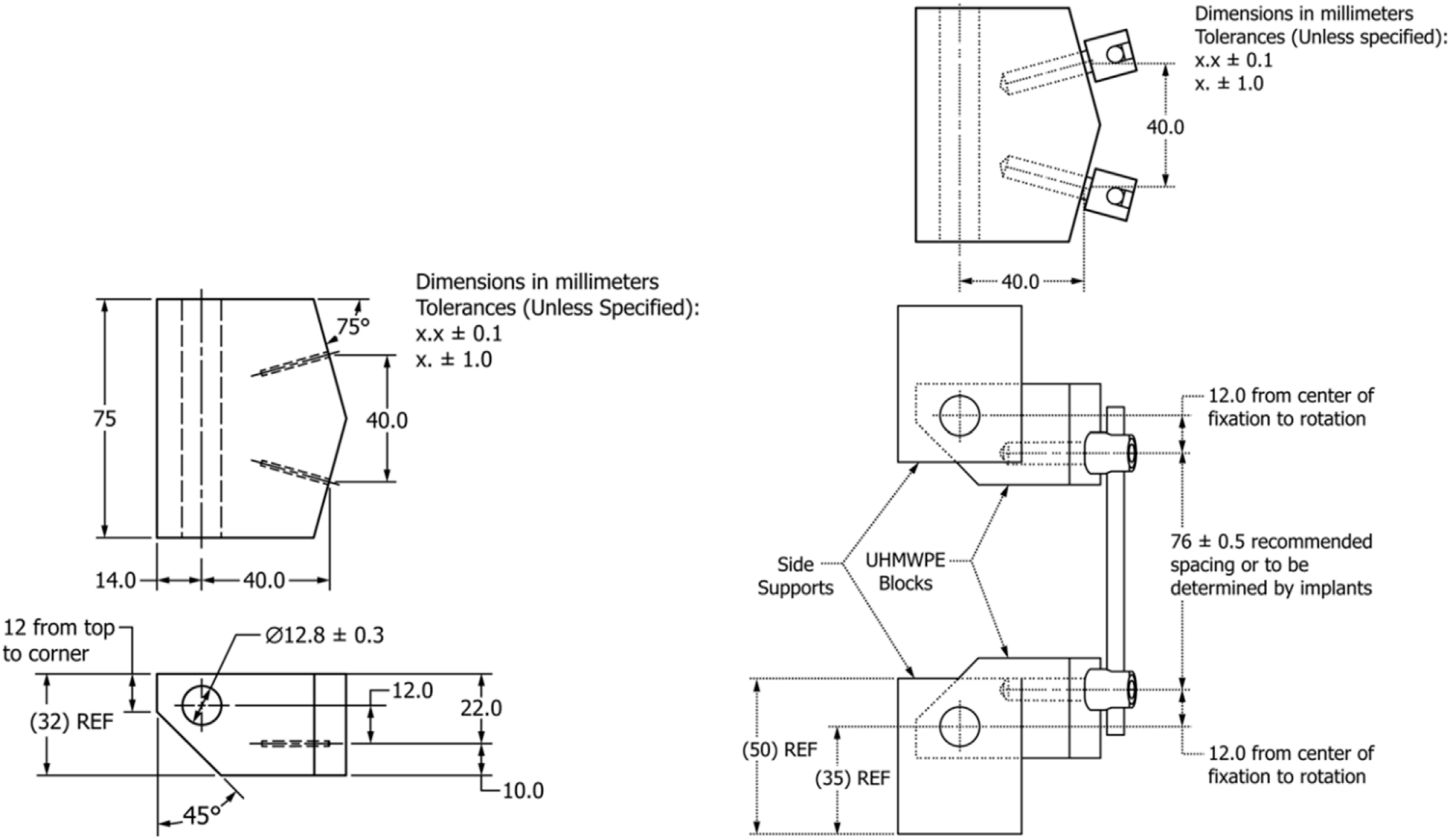
Design of UHMWPE blocks and experimental setup for spinal construct based on ASTM F1717 (ASTM International, 2009; ASTM International, 2015).

The spinal construct was assembled in accordance with the “Standard Test Methods for Spinal Implant Constructs in a Vertebrectomy Model” (ASTM International, 2009; ASTM International, 2015), and static tensile and compressive tests were conducted using a universal testing machine (UTM; AGS-X 1kN, Shimadzu, Kyoto, Japan) (Figure 1). Experimental data were automatically recorded using the software provided by the UTM manufacturer, and load–displacement data were collected at a sampling frequency of 100 Hz to precisely monitor the mechanical response of the construct during tensile and compressive testing. All tests were conducted on the same day in the same laboratory by the same operator, using the UTM described above.

**FIGURE 2:**
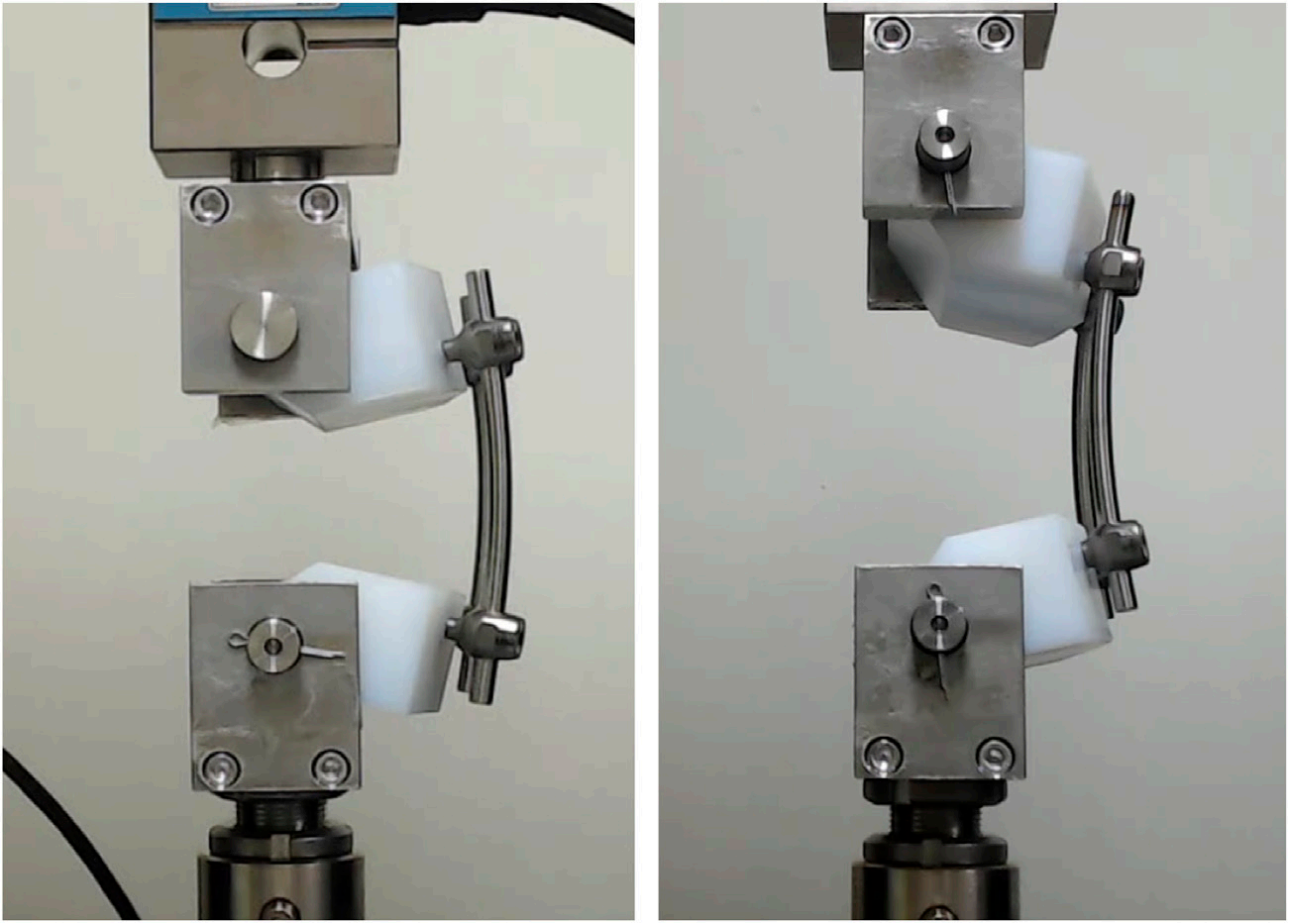
Static compression and tension tests.

The geometric parameters of the pedicle screws were set at diameters of 6 mm and 7 mm, and lengths of 40 mm and 50 mm, with the 50 mm screws designed to fully penetrate the UHMWPE block. In addition, pedicle screws with a diameter of 6 mm and length of 40 mm were designated as “640,” and the same naming convention was applied to all experimental groups. A total of eight experimental setups were tested in this study, consisting of four static compression tests (640, 650, 740, 750) and four static tension tests (640, 650, 740, 750). Each experimental group underwent five tests, as recommended by the ASTM F1717 standard for sample size, resulting in a total of 40 experimental runs. In accordance with ASTM F1717 standards, the loading rate was set to 25 mm/min, and tensile and compressive tests were performed up to a maximum displacement of 20 mm (Figure 2). The load–displacement curves obtained from the experiments were used to analyze the mechanical behavior of the spinal constructs, and key parameters such as elastic load, elastic displacement, stiffness at 2–10 mm displacement, yield force, yield displacement and load and displacement at 20 mm were recorded.

### 2.2 Finite element modeling and material properties

**FIGURE 3:**
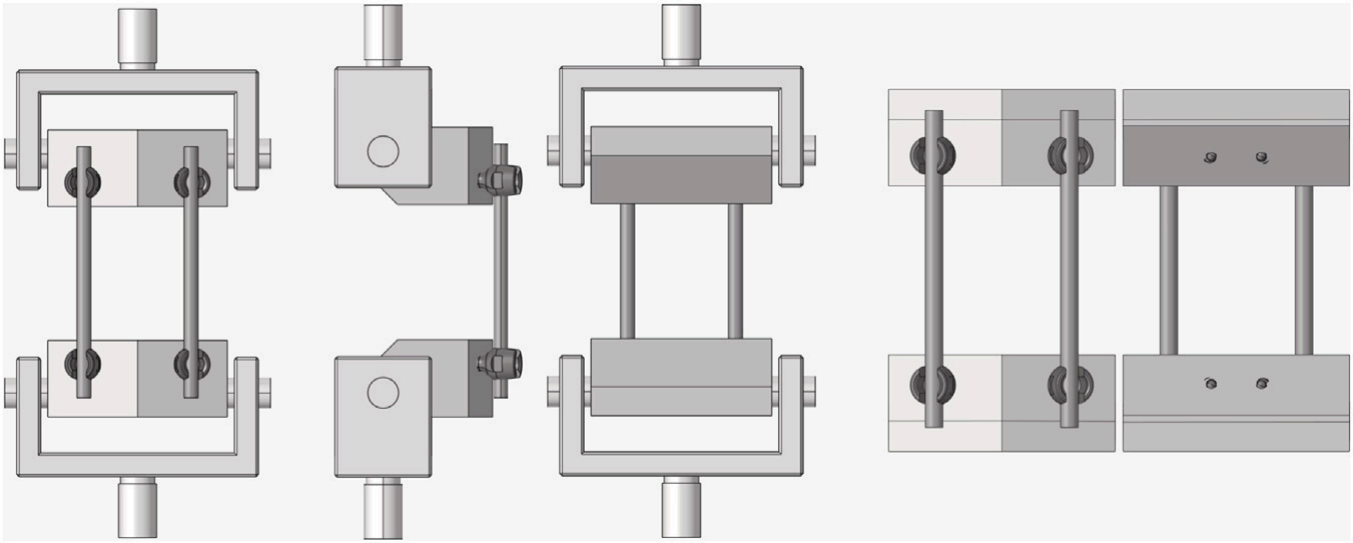
3D model of the spinal construct.

The finite element method is a numerical technique for analyzing complex structures and systems by discretizing the entire domain into smaller elements and obtaining approximate solutions. This method is widely utilized in various engineering fields including mechanical engineering, structural analysis, biomechanics, electromagnetics, fluid mechanics for structural, and thermal. In this study, a three-dimensional (3D) model representing the physical experimental setup was constructed to perform CM&S (Figure 3).

**FIGURE 4:**
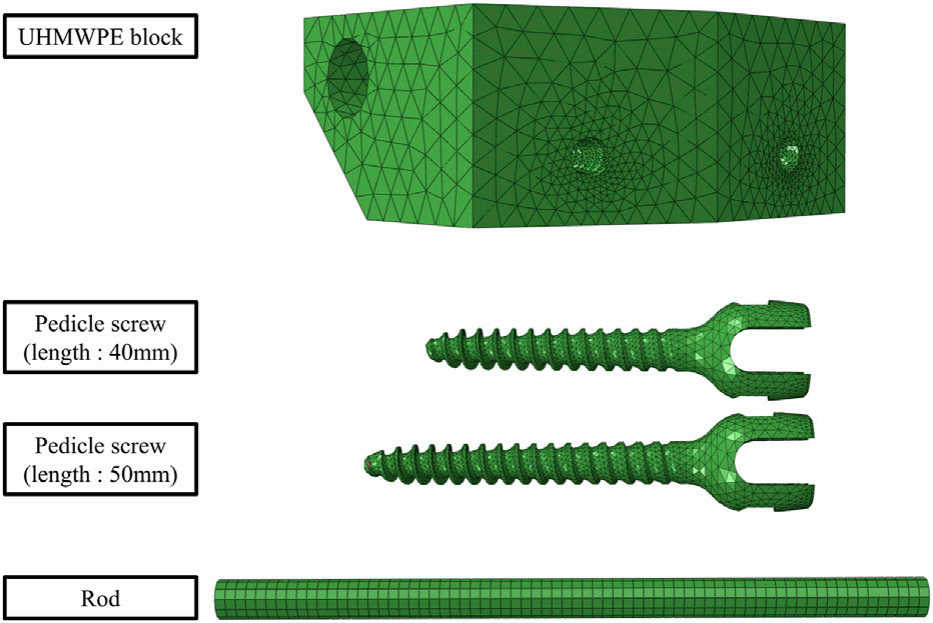
Finite element model of the spinal construct.

The UHMWPE blocks and spinal rods were modeled as three-dimensional solid bodies, and the pedicle screws were digitized by a professional 3D-scanning service (Freeismtech, Republic of Korea) using an optical structured-light workflow with multi-angle acquisition. The service used calibrated projection of coded light patterns with synchronized imaging to acquire dense point clouds, followed by view-to-view alignment and global registration to ensure geometric consistency. The generated models were meshed using Hypermesh 2024 (Altair Engineering Inc.). Considering the complex geometry, tetrahedral elements (C3D10) were applied to the pedicle screws and UHMWPE blocks, with each element containing 10 nodes to improve computational accuracy. The spinal rod, with its relatively simple geometry, was modeled using hexahedral elements (C3D8R) to enhance computational efficiency (Figure 4).

Table 1 summarizes the material properties used in the FEA, including elastic modulus, Poisson’s ratio, density, and yield strength for the pedicle screws, spinal rods, UHMWPE blocks, and jigs (Narita et al., 2012; https://www.azom.com/article.aspx?ArticleID=9365, Yucheng, 2009). The pedicle screws and spinal rods were assigned the material properties of Ti-6Al-4V ELI, and the UHMWPE blocks were characterized based on the stress–strain curve obtained from material testing conducted in accordance with ASTM D638-14 standards (ASTM International, 2014). Experimental results confirmed that the UHMWPE blocks satisfied the tensile strength requirement of 40 (±3) MPa specified in ASTM F1717 (ASTM International, 2015). The jig was used as an auxiliary fixture to support the spinal construct during testing, and since the mechanical response of the jig itself was not the focus of evaluation, its material properties were assigned up to the elastic region only. All materials were assumed to be isotropic in the finite element analysis.

**TABLE 1:** Material properties of jig, UHMWPE block, pedicle screw and spinal rod.

Materials	Density (ton/mm^3^)	Young’s modulus (MPa)	Poisson’s ratio	Yield stress (MPa)
Jig (mild steel)	7.83e-009	207,000	0.3	-
UHMWPE block	9.30e-010	282	0.46	-
Pedicle screw & spinal rod (Ti6Al4V-ELI)	4.429e-009	108,000	0.342	864

### 2.3 Mesh convergence study, boundary conditions, and loading conditions

To ensure the reliability and accuracy of the FEA, we first conducted a mesh-convergence study as solution verification to quantify and control discretization error as the mesh was refined, then selected the optimal mesh size. Simulations were performed in Abaqus 2024 (Dassault Systèmes SIMULIA, USA). This procedure helped to ensure numerical precision and reproducibility; Table 2 summarizes the convergence metrics and the final mesh settings.

**TABLE 2:** Element refinement conditions for discretization error verification.

No	Mesh size (mm)	No. of elements
1	0.1	427,403
2	0.2	413,807
3	0.3	394,044
4	0.4	374,904
5	0.5	303,297
6	0.7	220,250
7	0.9	167,590
8	1	144,147
9	1.5	104,970
10	2	95,910
11	3	88,846
12	5	77,305

**FIGURE 5:**
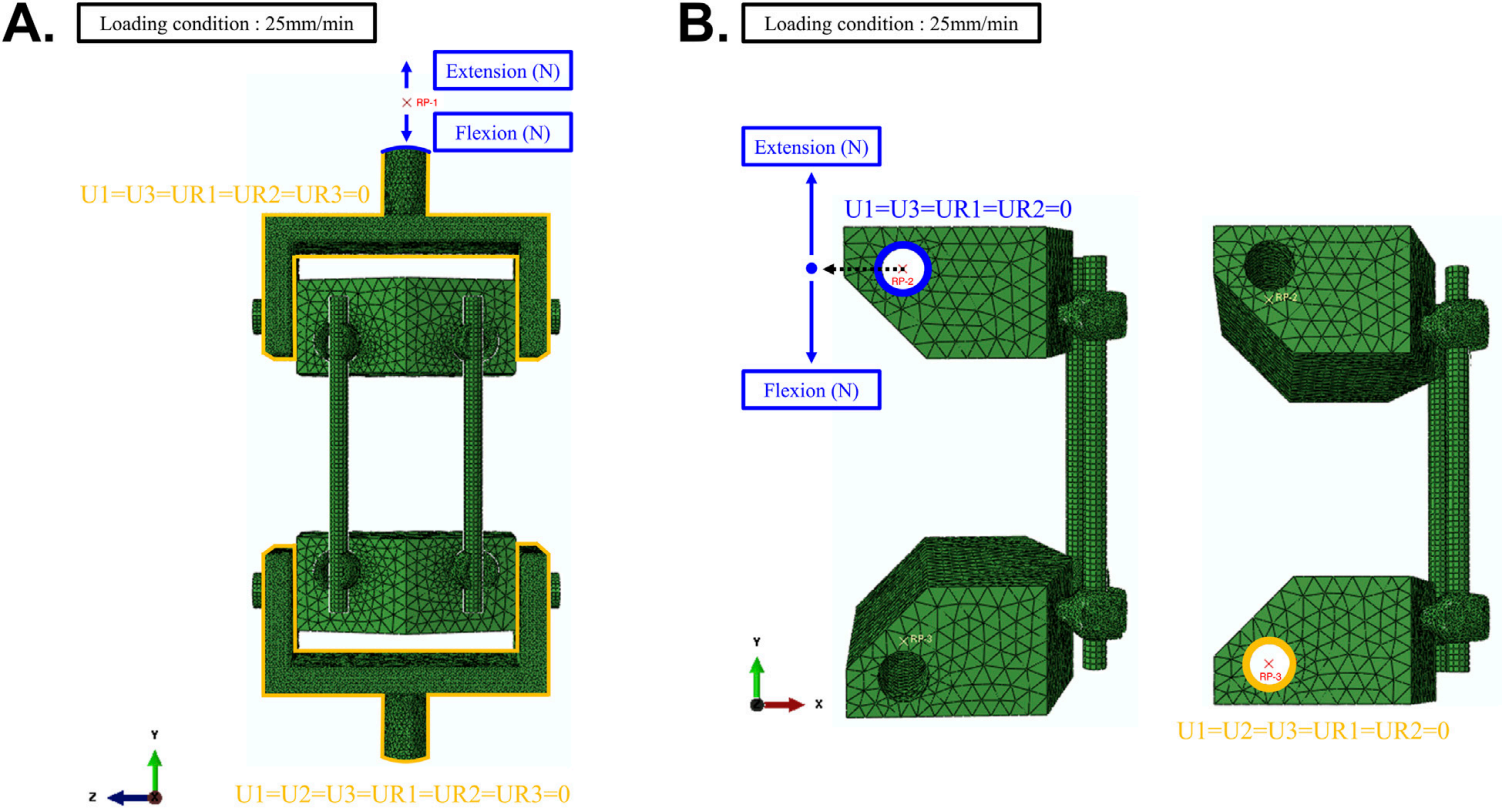
**(A)** Boundary and loading conditions in FEA including jig, **(B)** Boundary and loading conditions in FEA excluding jig.

Subsequently, finite element analyses (FEA) of the spinal construct were performed under two conditions. As a VVUQ-oriented model form investigation, we assessed the impact of jig omission by comparing FEA models with and without the jig under static compression and tension, and quantified the effect on computational time. The jig-included model and jig-excluded model are denoted JIM and JEM, respectively. The first condition (JIM) included the jig to closely replicate the physical testing setup, whereas the second condition (JEM) excluded the jig and modified the boundary and loading conditions to enhance computational efficiency. Figure 5A illustrates the boundary and loading conditions applied in the FEA of this study. In the JIM analysis, the lower jig was fully fixed in all directions, while the upper jig was constrained in all degrees of freedom except for translation along the y-axis. A reference point (RP-1) was established at a vertical distance from the top surface of the jig to apply boundary conditions and loading through a coupling constraint (Figure 5A).

In the JEM analysis, to enhance computational efficiency and simplify boundary condition application, a reference point (RP-3) was placed at the midpoint inside the cylindrical insertion area of the lower UHMWPE block (Figure 5B). This RP was connected to the cylindrical surface through a coupling constraint, and all degrees of freedom except for rotation along the z-axis were restricted. Similarly, the upper UHMWPE block was assigned a reference point (RP-2), where all degrees of freedom were constrained except for z-axis rotation and y-axis translation. A concentrated load was applied to the upper RP to perform both compression and tension tests. Additionally, tie constraints were assumed between the UHMWPE and pedicle screws, and between the pedicle screws and spinal rods, for all analyses. After comparing the two simulation setups, the more efficient method was selected, and its results were analyzed in detail.

## 3 Results

### 3.1 Experimental results of the spinal construct

The spinal construct was assembled by combining pedicle screws, spinal rods, and UHMWPE blocks as shown in Figure 1, and mounted onto a UTM. Subsequently, static compression and tension tests were conducted at a loading rate of 25 mm/min in accordance with ASTM F1717 standards (ASTM International, 2009; ASTM International, 2015). Table 3 presents the elastic load, elastic displacement, stiffness, and force at 20 mm displacement under static compression loading for each pedicle screws (640, 650, 740, 750). Under static compression, the 640 group showed an elastic load of 685.75 ± 82.43 N, an elastic displacement of 15.00 ± 2.32 mm, a stiffness of 48.35 ± 2.24 N/mm, and a force at 20 mm displacement of 864.22 ± 20.91 N. The 650 group exhibited 678.15 ± 67.44 N, 14.94 ± 1.88 mm, 47.91 ± 1.86 N/mm, and 847.97 ± 29.93 N, respectively. The 740 group recorded 764.94 ± 57.64 N, 14.80 ± 1.26 mm, 54.45 ± 1.40 N/mm, and 932.62 ± 18.58 N. Finally, the 750 group exhibited 721.73 ± 30.77 N, 14.60 ± 0.54 mm, 52.03 ± 1.08 N/mm, and 888.72 ± 22.12 N. Table 4 presents the results under static tension. For the 640 group, the elastic load was 967.07 ± 88.16 N, the elastic displacement was 18.98 ± 1.02 mm, the stiffness was 54.55 ± 2.27 N/mm, and the force at 20 mm displacement was 1,032.93 ± 56.14 N. The 650 group showed a stiffness of 57.70 ± 2.46 N/mm and a force at 20 mm displacement of 1,049.44 ± 42.57 N. In the 740 group, the elastic load averaged 1,082.42 ± 47.20 N, the elastic displacement was 17.98 ± 0.54 mm, the stiffness was 63.38 ± 1.99 N/mm, and the force at 20 mm displacement was 1,162.94 ± 36.40 N. Finally, the 750 group exhibited 1,001.21 ± 29.69 N, 17.09 ± 0.83 mm, 61.73 ± 1.78 N/mm, and 1,132.60 ± 29.19 N.

**TABLE 3:** Static compression test results.

No.	Elastic load (N)	Elastic displacement (mm)	Stiffness (N/mm)	Yield force (N)	Yield displacement (mm)	Force at 20 mm (N)
640-1	626.08	12.94	50.91	778.31	16.81	877.07
640-2	789.18	17.62	47.14	832.43	18.82	871.00
640-3	649.36	14.71	46.45	783.60	18.38	832.75
640-4	758.11	17.12	46.60	843.00	19.60	854.69
640-5	606.01	12.59	50.66	802.80	17.36	885.58
640 average	685.75 (±82.43)	15.00 (±2.32)	48.35 (±2.24)	808.03 (±28.83)	18.19 (±1.12)	864.22 (±20.91)
650-1	610.65	13.72	46.85	727.20	17.03	812.19
650-2	671.99	14.09	50.20	804.41	17.54	876.86
650-3	622.52	13.23	49.54	817.66	18.06	881.17
650-4	710.02	15.86	47.10	783.20	18.14	831.63
650-5	775.55	17.79	45.87	825.19	19.51	837.98
650 average	678.15 (±67.44)	14.94 (±1.88)	47.91 (±1.86)	791.53 (±39.34)	18.06 (±0.93)	847.97 (±29.93)
740-1	705.10	14.04	52.87	814.00	16.91	902.14
740-2	722.49	13.58	56.01	857.20	16.82	945.14
740-3	766.32	14.79	54.54	850.00	17.10	929.84
740-4	777.86	14.73	55.61	857.00	16.92	936.99
740-5	852.91	16.87	53.22	912.20	18.65	948.98
740 average	764.94 (±57.64)	14.80 (±1.26)	54.45 (±1.40)	858.08 (±35.14)	17.28 (±0.77)	932.62 (±18.58)
750-1	704.73	14.62	50.75	780.20	16.89	859.25
750-2	696.10	13.91	52.66	811.80	16.93	899.02
750-3	715.38	14.77	50.97	801.60	17.24	873.89
750-4	774.52	15.38	53.02	852.00	17.59	915.31
750-5	717.90	14.33	52.73	810.20	16.88	896.15
750 average	721.73 (±30.77)	14.60 (±0.54)	52.03 (±1.08)	811.16 (±26.07)	17.11 (±0.31)	888.72 (±22.12)

**TABLE 4:** Static tension test results.

No.	Elastic load (N)	Elastic displacement (mm)	Stiffness (N/mm)	Yield force (N)	Yield displacement (mm)	Force at 20 mm (N)
640-1	976.34	19.59	52.45	-	-	990.97
640-2	874.65	17.80	51.73	955.88	20.00	955.88
640-3	-	-	55.95	-	-	1,083.86
640-4	-	-	56.02	-	-	1,067.61
640-5	1,050.23	19.54	56.58	-	-	1,066.31
640 average	967.07 (±88.16)	18.98 (±1.02)	54.55 (±2.27)	-	-	1,032.93 (±56.14)
650-1	-	-	51.84	-	-	997.62
650-2	-	-	53.03	-	-	1,028.82
650-3	-	-	55.67	-	-	1,061.17
650-4	-	-	54.77	-	-	1,046.95
650-5	-	-	58.20	-	-	1,112.63
650 average	-	-	54.70 (±2.46)	-	-	1,049.44 (±42.57)
740-1	1,043.15	18.17	60.43	-	-	1,118.33
740-2	1,098.57	18.38	62.92	1,161.66	20.00	1,161.66
740-3	1,157.47	18.53	65.76	-	-	1,218.09
740-4	1,048.48	17.42	63.34	1,120.23	19.20	1,147.76
740-5	1,064.45	17.38	64.47	1,140.30	19.20	1,168.86
740 average	1,082.42 (±47.20)	17.98 (±0.54)	63.38 (±1.99)	1,140.73 (±20.72)	19.47 (±0.46)	1,162.94 (±36.40)
750-1	994.43	17.69	59.16	1,071.93	19.63	1,183.88
750-2	1,008.54	17.30	61.36	1,083.83	19.18	1,112.29
750-3	989.63	16.45	63.32	1,067.50	18.37	1,125.01
750-4	1,046.93	17.97	61.32	1,116.97	19.73	1,125.12
750-5	966.53	16.02	63.51	1,047.20	18.00	1,116.69
750 average	1,001.21 (±29.69)	17.09 (±0.83)	61.73 (±1.78)	1,077.49 (±25.72)	18.98 (±0.77)	1,132.60 (±29.19)

**FIGURE 6:**
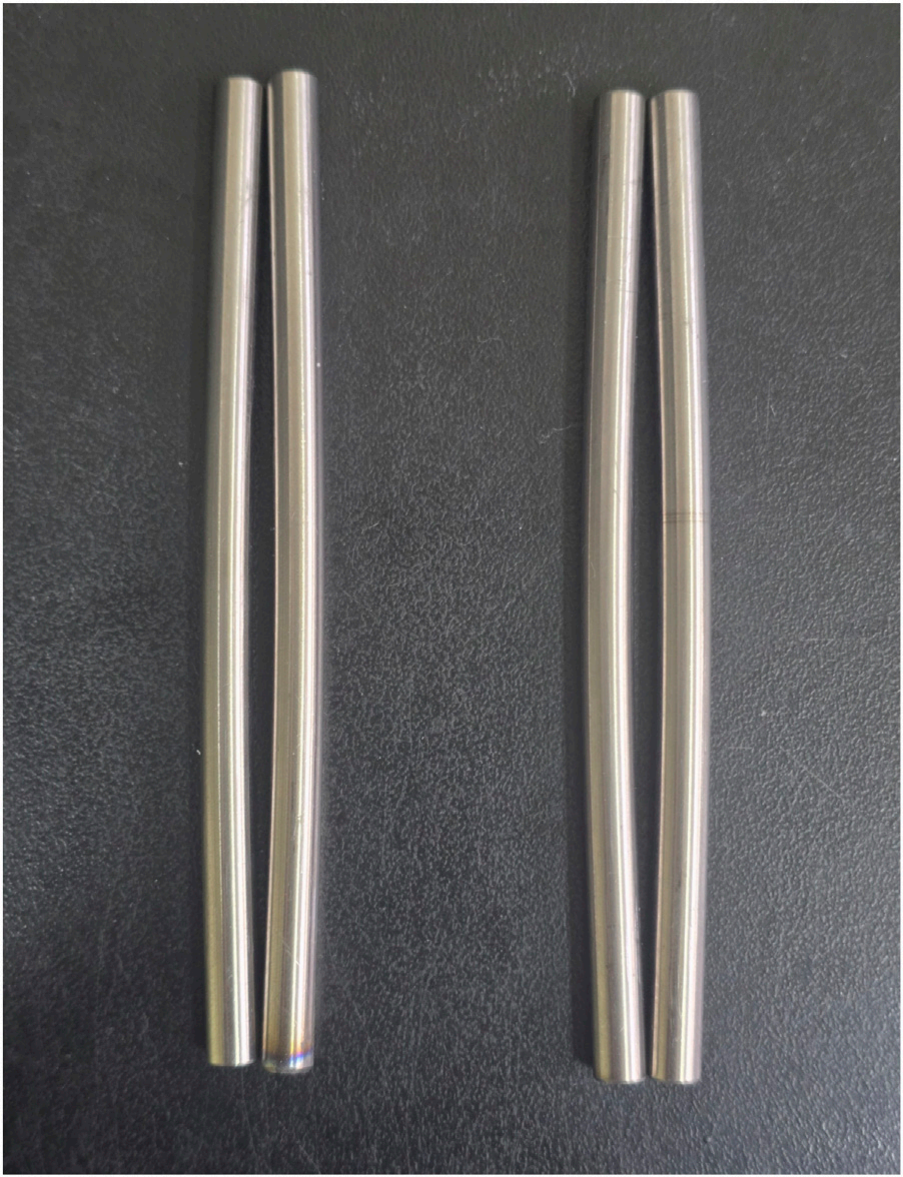
Permanently deformed spinal rods after 20 mm displacement in static compression and tension tests.

Upon visual inspection after completing the static compression and tension tests, no noticeable deformation was observed in the pedicle screws or tightening screws. However, permanent deformation of the spinal rods was observed at 20 mm displacement under both compression and tension conditions (Figure 6).

### 3.2 FEA results of the spinal construct

In this study, a mesh convergence study was conducted to ensure the accuracy and reliability of the FEA. The reaction force at a displacement of 20 mm was compared across different mesh sizes, and convergence was considered achieved when the change rate between successive results was within 1%. It was confirmed that the reaction force variation across all mesh sizes was within 1%, and a mesh size demonstrating clear stabilization of the results was selected. As a result, a mesh size of 0.4 mm was determined, showing a reaction force convergence rate of 0.01% and stabilizing at 830.30 N. Meanwhile, other mesh sizes showed variation rates ranging from 0.3% to 0.03% (Table 5). This process allowed for the selection of an optimal mesh size that ensured both computational efficiency and solution accuracy.

**TABLE 5:** Discretization error verification.

No	Mesh size (mm)	No. of elements	Reaction force (N, at 20 mm)
1	0.1	427,403	830.29
2	0.2	413,807	830.30
3	0.3	394,044	830.32
4	0.4	374,904	830.36
5	0.5	303,297	830.46
6	0.7	220,250	831.23
7	0.9	167,590	832.06
8	1	144,147	832.34
9	1.5	104,970	834.93
10	2	95,910	835.83
11	3	88,846	836.76
12	5	77,305	838.52

Based on the selected mesh size, static compression and tension analyses of the spinal construct were performed under two configurations: JIM and JEM, as shown in Figure 5. In static compression, adopting JEM reduced computational time by approximately 21.26%–31.27%, while the error in reaction force at 20 mm displacement remained low at 0.14%–0.19%. Similarly, in static tension, JEM reduced computational time by 20.51%–32.22% and maintained a reaction force error within 0.17%–0.24% (Table 6).

**TABLE 6:** Comparison of FEA results JIM and JEM.

Test mode	Case	JIM		JEM		Reduced time (%)	Error rate (%)
		The time required (min)	Reaction force (N, at 20 mm)	The time required (min)	Reaction force (N, at 20 mm)		
Flexion	640	311.53	831.71	214.11	830.36	31.27	0.16
	650	383.78	841.59	284.93	842.86	25.75	0.15
	740	411.86	867.51	324.28	866.25	21.26	0.14
	750	517.41	872.00	369.10	870.26	28.66	0.19
Extension	640	308.79	1,074.84	209.25	1,076.72	32.22	0.17
	650	398.71	1,092.38	282.75	1,095.08	29.08	0.24
	740	409.30	1,123.26	325.35	1,125.36	20.51	0.18
	750	526.36	1,127.59	387.83	1,129.53	26.32	0.17

**FIGURE 7:**
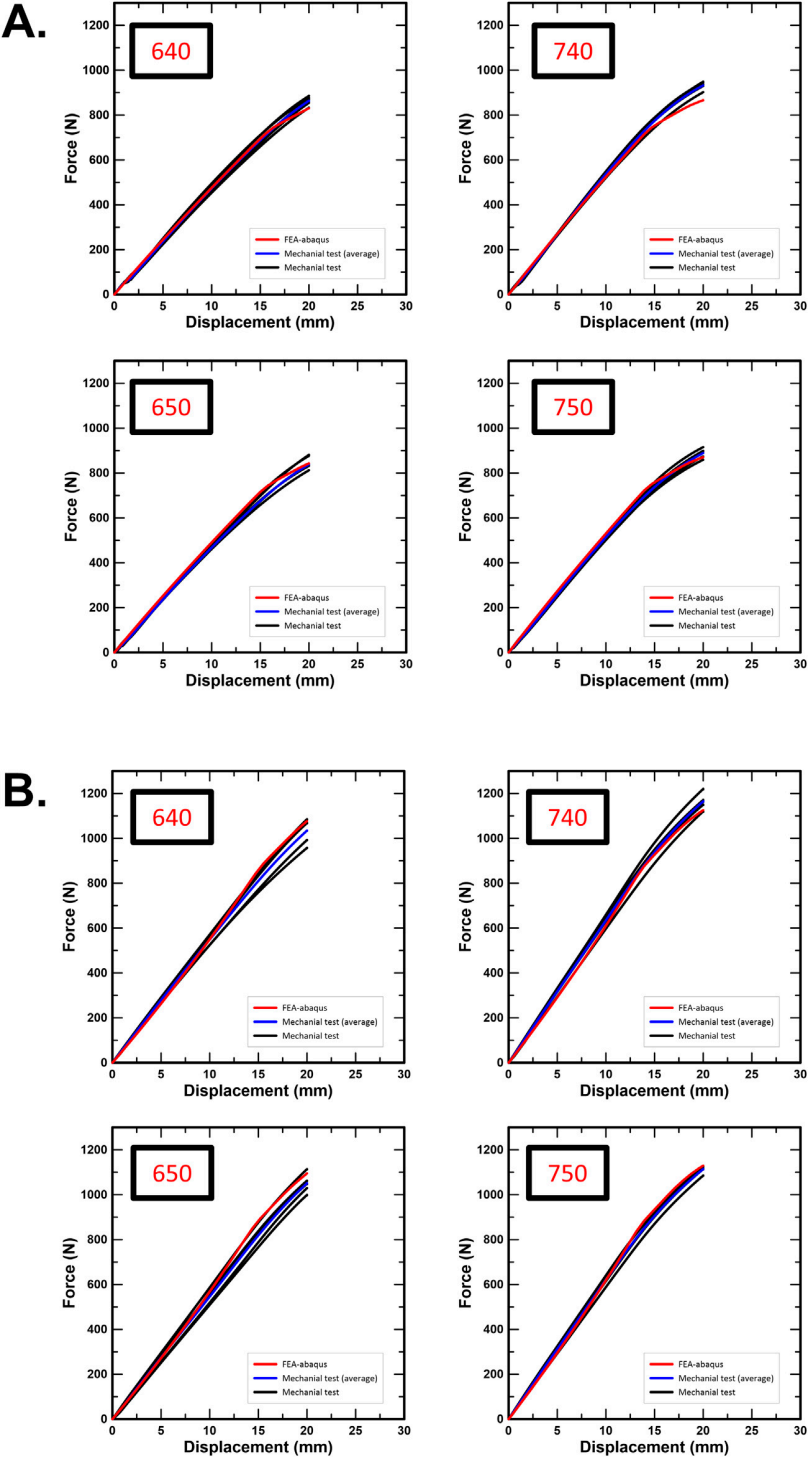
**(A)** Force-displacement curve from static compression test of the spinal construct, **(B)** Force-displacement curve from static tension test of the spinal construct.

To validate the FEA results, simulations were conducted corresponding to the conditions of the physical experiments based on ASTM F1717, and force-displacement (F-D) curves were generated for comparison, as shown in Figure 7. Visual inspection revealed that the FEA-generated F-D curves closely followed the overall trends observed in the physical tests. To quantitatively assess the agreement, Table 7 summarizes the reaction force values at 20 mm displacement and their corresponding error rates between experimental and FEA results.

**TABLE 7:** Experiment–FEA comparison: force at 20 mm, stiffness, yield (flexion/extension).

Test mode	Result type	Stiffness (N/mm)	Yield force (N)	Yield displacement (mm)	Force at 20 mm (N)
Flexion	640 experiment average	48.35 (±2.24)	808.03 (±28.83)	18.19 (±1.12)	864.22 (±20.91)
	Abaqus	47.00	795.40	18.44	830.36
	Error-average (%)	2.80	1.57	1.37	3.92
	650 experiment average	47.91 (±1.86)	791.53 (±39.34)	18.06 (±0.93)	847.97 (±29.93)
	Abaqus	48.43	800.28	18.04	842.86
	Error-average (%)	1.07	1.11	0.11	0.60
	740 experiment average	54.45 (±1.40)	858.08 (±35.14)	17.28 (±0.77)	932.62 (±18.58)
	Abaqus	51.73	807.42	17.13	866.25
	Error-average (%)	4.99	5.90	0.87	7.12
	750 experiment average	52.03 (±1.08)	811.16 (±26.07)	17.11 (±0.31)	888.72 (±22.12)
	Abaqus	52.35	808.34	17.11	870.26
	Error-average (%)	0.61	0.35	0.88	2.08
Extension	640 experiment average	54.55 (±2.27)	-	-	1,032.93 (±56.14)
	Abaqus	55.90	1,137.3	21.87	1,076.72
	Error-average (%)	2.47	-	-	4.24
	650 experiment average	54.70 (±2.46)	-	-	1,049.44 (±42.57)
	Abaqus	57.78	1,127.55	21.03	1,095.08
	Error-average (%)	5.63	-	-	4.35
	740 experiment average	63.38 (±1.99)	1,140.73 (±20.72)	19.47 (±0.46)	1,162.94 (±36.40)
	Abaqus	62.10	1,105.01	19.47	1,125.36
	Error-average (%)	2.01	3.13	0.82	3.23
	750 experiment average	61.73 (±1.78)	1,077.49 (±25.72)	18.98 (±0.77)	1,132.60 (±29.19)
	Abaqus	62.93	1,100.75	18.98	1,129.53
	Error-average (%)	1.94	2.16	0.15	0.27

Under flexion, the 640 group showed an experimental stiffness of 48.35 ± 2.24 N/mm versus an FEA value of 47.00 N/mm (error 2.80%); a yield force of 808.03 ± 28.83 N versus 795.40 N (1.57%); a yield displacement of 18.19 ± 1.12 mm versus 18.44 mm (1.37%); and a force at 20 mm of 864.22 ± 20.91 N versus 830.36 N (3.92%). For the 650 group, stiffness was 47.91 ± 1.86 N/mm versus 48.43 N/mm (1.07%); yield force 791.53 ± 39.34 N versus 800.28 N (1.11%); yield displacement 18.06 ± 0.93 mm versus 18.04 mm (0.11%); and force at 20 mm 847.97 ± 29.93 N versus 842.86 N (0.60%). For the 740 group, stiffness was 54.45 ± 1.40 N/mm versus 51.73 N/mm (4.99%); yield force 858.08 ± 35.14 N versus 807.42 N (5.90%); yield displacement 17.28 ± 0.77 mm versus 17.13 mm (0.87%); and force at 20 mm 932.62 ± 18.58 N versus 866.25 N (7.12%). For the 750 group, stiffness was 52.03 ± 1.08 N/mm versus 52.35 N/mm (0.61%); yield force 811.16 ± 26.07 N versus 808.34 N (0.35%); yield displacement 17.11 ± 0.31 mm versus 17.11 mm (0.88%); and force at 20 mm 888.72 ± 22.12 N versus 870.26 N (2.08%).

Under extension, the 640 group showed stiffness of 54.55 ± 2.27 N/mm versus 55.90 N/mm (2.47%) and a force at 20 mm of 1,032.93 ± 56.14 N versus 1,076.72 N (4.24%). For the 650 group, stiffness was 54.70 ± 2.46 N/mm versus 57.78 N/mm (5.63%) and the force at 20 mm was 1,049.44 ± 42.57 N versus 1,095.08 N (4.35%). For the 740 group, stiffness was 63.38 ± 1.99 N/mm versus 62.10 N/mm (2.01%); yield force 1,140.73 ± 20.72 N versus 1,105.01 N (3.13%); yield displacement 19.47 ± 0.46 mm versus 19.47 mm (0.82%); and force at 20 mm 1,162.94 ± 36.40 N versus 1,125.36 N (3.23%). For the 750 group, stiffness was 61.73 ± 1.78 N/mm versus 62.93 N/mm (1.94%); yield force 1,077.49 ± 25.72 N versus 1,100.75 N (2.16%); yield displacement 18.98 ± 0.77 mm versus 18.98 mm (0.15%); and force at 20 mm 1,132.60 ± 29.19 N versus 1,129.53 N (0.27%). Overall, FEA reproduced each metric within errors generally below 5%, with the largest deviation of 7.12% observed for the flexion force at 20 mm in the 740 group.

**FIGURE 8:**
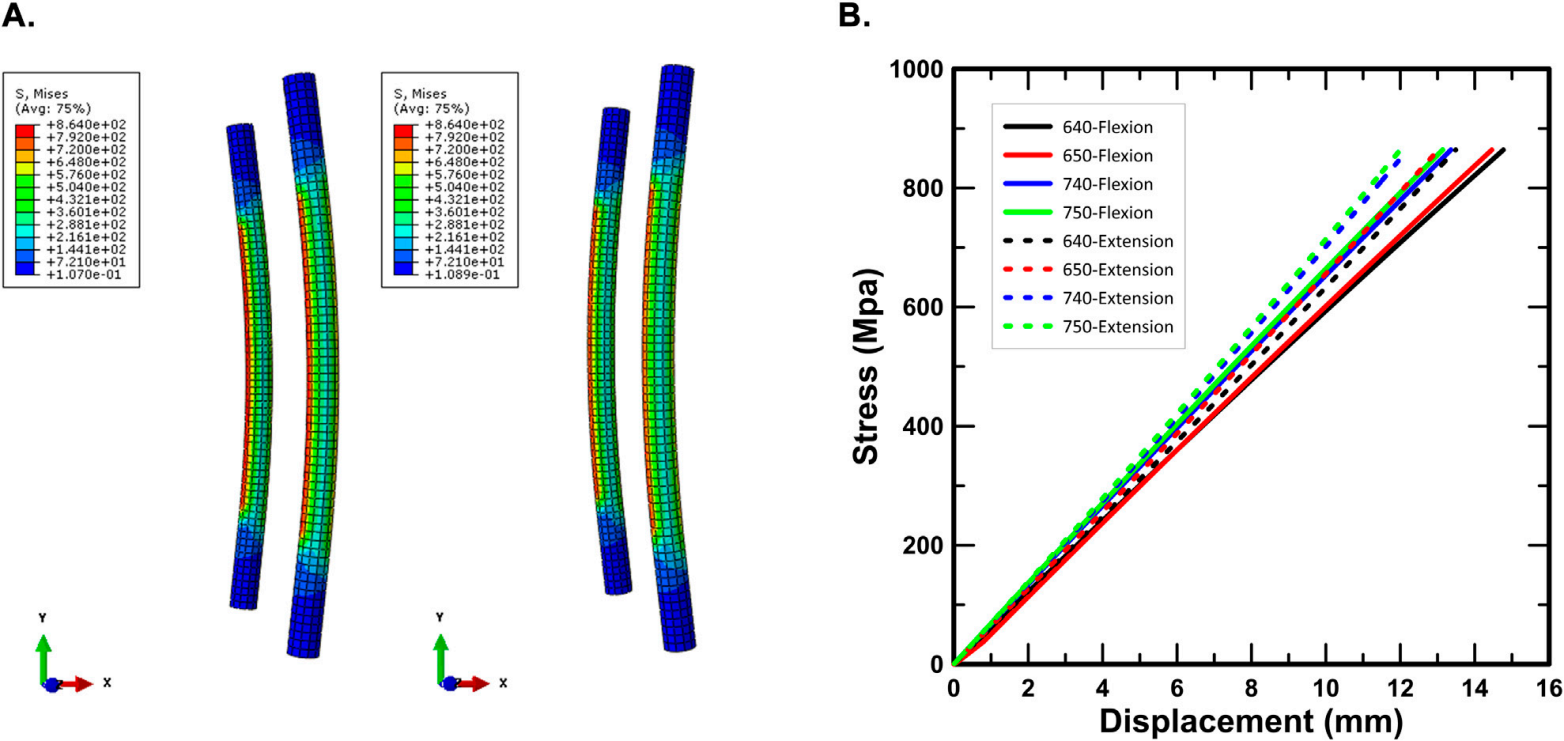
Yield behavior of spinal rods in FEA: **(A)** deformed shape of the rod at yield; **(B)** construct displacement at yield.

Figure 8 illustrates the yielding behavior of the spinal rod under both static compression and tension loading conditions based on FEA results. The yielding displacements under static compression loading were observed at 14.77 mm (640 case), 14.46 mm (650 case), 13.36 mm (740 case), and 13.14 mm (750 case). Under static tension loading, the yielding displacements were found to be 13.49 mm (640 case), 13.02 mm (650 case), 12.20 mm (740 case), and 12.00 mm (750 case).

## 4 Discussion

This study aimed to evaluate the static mechanical behavior of spinal fixation constructs based on the ASTM F1717 standard and to assess the feasibility of using CM&S for simulation-based design and structural validation through comparison with experimental data. To this end, spinal constructs were assembled using pedicle screws, spinal rods, and UHMWPE blocks, and force–displacement curves were acquired using a UTM. A parametric analysis was performed by varying the pedicle screw type (640, 650, 740, 750), and key mechanical metrics including stiffness, elastic load, elastic displacement, and reaction force at 20 mm displacement were quantitatively compared across both experimental and simulation results. To define the thresholds of elastic behavior, the initial 0–2 mm displacement region was excluded due to the potential for experimental errors such as microgaps or unstable initial contact, even in well-tightened constructs. Consequently, the stiffness calculated at 2–10 mm displacement was considered representative of the elastic region, and all tests confirmed linear behavior in this range. The upper bounds of elastic load and displacement were defined at the point where the stiffness error exceeded 5%, marking the end of the linear response on the force–displacement (F–D) curve. This approach allowed the identification of the elastic-to-plastic transition zone in a quantifiable manner. The physical testing results showed relatively low standard deviations for stiffness, indicating high consistency and reliability. The physical tests showed a low standard deviation in stiffness, indicating high consistency and reliability. Stiffness, defined as the slope of the load-displacement curve within the elastic region, is a representative linear metric. At the maximum displacement used in this study (20 mm), the reaction force also exhibited low variability, supporting its consistency and reliability. The reaction force at 20 mm provides an intuitive measure of the construct’s ability to sustain a specified deformation and enables clear, single-value comparisons across screw configurations. In contrast, stiffness is a useful comparator but is relatively sensitive to nonlinearity caused by seating effects and the closure of microgaps in the 0–2 mm region. Therefore, we selected the reaction force at 20 mm as the primary comparative metric, complemented by stiffness, yield load, and yield displacement.

To ensure the reliability and accuracy of the FEA results, a mesh convergence study was first conducted. The convergence behavior was evaluated based on the reaction force at a displacement of 20 mm across various mesh sizes. As a result, the change in reaction force remained within 1% across all mesh sizes, indicating that the model exhibited numerical stability. Specifically, at a mesh size of 0.4 mm, the variation was observed to be only 0.01%, with a reaction force converging at 830.36 N, demonstrating convergence to the target value of 830.30 N. This result was interpreted as achieving an optimal balance between computational efficiency and numerical accuracy, and thus the mesh size of 0.4 mm was subsequently adopted for all further analyses.

Subsequently, the effects of JEM on FEA computational time were investigated under static compression and tensile loading conditions applied to the spinal fixation constructs. In the compression analysis, JEM reduced the computational time by up to 31.27%, while the error rate in reaction force at 20 mm displacement remained as low as 0.19%. Similarly, under tensile loading, computational time decreased by up to 32.22% with JEM, with reaction force error rates within 0.24%. These findings suggest that the JEM simplification strategy introduces minimal impact on simulation accuracy, while significantly improving computational efficiency. Notably, the reaction force error rates remained well within the generally acceptable engineering margin of 5%, and even under a stricter 1% threshold, the results maintained sufficient reliability. Thus, the mesh convergence verification combined with JEM is interpreted as a practical strategy for achieving an optimal balance between simulation accuracy and efficiency, and can be effectively applied to future iterative design evaluations or parametric studies.

Furthermore, the experimental and FEA results for the spinal fixation constructs were visualized using force–displacement (F–D) curves. Although visual inspection indicated general similarity between experimental and simulation results, a quantitative validation was essential to assess the practical applicability of the simulation. Thus, the reaction force at a displacement of 20 mm, previously validated during mesh convergence and jig simplification processes, was adopted as the quantitative comparison metric. This displacement point was considered to comprehensively capture the linear, nonlinear, and yielding mechanical responses of the construct. Consistent with this, visual inspection (Figure 6) confirmed the presence of permanent deformation of the spinal rod within 20 mm of displacement, implying that the structural system had reached the mechanical limit transitioning into plastic deformation. Therefore, the reaction force at 20 mm was determined as a valid and meaningful criterion for evaluating structural durability and design safety between experimental and simulation results. Furthermore, considering that a single spinal segment clinically allows physiological motion of less than 16 mm, a complete system accommodating two spinal gaps would experience less than 32 mm of displacement under extreme loading conditions (Yucheng, 2009; Dvorák et al., 1991). However, in this study, the spinal rod exhibited clear plastic deformation well before reaching a displacement of 20 mm. Thus, displacements beyond this point would not provide additional meaningful mechanical information but would instead enter an irreversible failure mode. Accordingly, this study determined that using the reaction force at 20 mm displacement serves as an appropriate comparative indicator for evaluating the structural durability and design safety of the spinal fixation system through simulation–experiment correlation.

Under static compression, the percent error between experimental and FEA results was generally maintained below 5%. An exception was the 740 group, which exhibited an error of 7.12%. This deviation likely reflects the accumulation of minor initial gaps and tightening inconsistencies introduced during the bench setup. Notably, the 740 group also tended to show a higher reaction force at 20 mm displacement, as well as higher yield stress and stiffness, which may stem from slight variations in material properties or manufacturing-induced inconsistencies. Under static tensile loading, agreement between experimental and FEA results was favorable, with errors below 5% in nearly all groups. These findings suggest that the FEA model replicates tensile-loading behavior more precisely than compressive-loading behavior.

The FEA results for the yielding displacement of the spinal rod indicated that yielding occurred between 13.14 and 14.77 mm under static compression loading and between 12.00 and 13.49 mm under static tensile loading (Figure 8). These findings suggest that plastic deformation of the spinal rod initiates well within the 20 mm simulation range, corroborating the permanent deformation observed in post-experimental inspections. Moreover, a tendency for shorter yielding displacements was observed as the screw diameter and length increased, which can be attributed to the enhanced fixation force leading to reduced load distribution capability and stress concentration on the spinal rod. As a result, the spinal rod reaches the yield stress within a shorter displacement range, and the observed trend of decreasing yield displacement corresponds precisely to the expected structural mechanics mechanism. This consistency between the experimental observations and theoretical interpretation reinforces the reliability of the simulation and the validity of the mechanical analysis approach employed in this study.

In this study, a systematic Model Risk assessment was conducted based on the ASME V&V 40-2018 guideline to evaluate the credibility and applicability of the developed FEA model [V&V 40–2018, 2018]. The Model Influence was assessed not as a simple reference, but rather as a critical factor intended to directly support design decision-making, especially considering the objective of replacing physical testing with FEA results. Therefore, the Model Influence was determined to fall at the boundary between medium (B) and high (C) levels, classified as B-C. Meanwhile, the Decision Consequence was set at a medium (B) level, reflecting that although incorrect model predictions would not directly cause patient harm, they could potentially necessitate revision surgeries or compromise the functional stability and design reliability of the implant system. The detailed definitions and classification criteria for these assessments are provided in Supplementary Table 1 and Figure 1 (See Supplementary Figure 1; Table 2). Overall, the FEA model established in this study was characterized by a Model Influence at the B-C level and a Decision Consequence at the B level, indicating an overall Model Risk ranging from medium to high. According to this risk classification, the required Validation Level was determined to be approximately Level 3-4. In the actual validation results, as previously described, most groups demonstrated quantitative error rates within 5% for reaction force comparisons between simulation and experimental data, with the 740 groups compression showing the highest discrepancy at 7.12%. This outcome satisfies our pre-specified acceptance criterion (≤10%) for this validation activity, which we aligned with an example Level-3 scope within the V&V 40 risk-informed credibility framework; V&V 40 does not prescribe universal numerical thresholds (American Society of Mechanical Engineers, 2018). The result supports the intended credibility for the model’s “Context of Use”. Consequently, it is concluded that the FEA developed in this study is sufficiently validated as a CM&S-based structural verification tool and can be effectively utilized as an efficient substitute for physical testing.

This study also proposes an optimized specification for the diameter and length of pedicle screws, based on the findings from both FEA and physical experiments, with the aim of informing more effective spinal fixation construct designs in clinical practice. To this end, changes in reaction force were quantitatively analyzed under static compression and tensile loading conditions. Under static compression, physical experiments showed that increasing the screw length from 640 to 650 decreased the reaction force by 1.88%, and from 740 to 750 by 4.71%. Conversely, increasing the screw diameter resulted in a reaction force increase of 7.91% from 640 to 740, and 4.81% from 650 to 750. Under static tensile loading, increasing the length from 640 to 650 led to a 1.60% increase, whereas from 740 to 750 it caused a 2.61% decrease. Diameter increases under tension resulted in an 12.59% increase from 640 to 740 and a 7.92% increase from 650 to 750. FEA results under the same conditions showed similar trends. Under compression, increasing the length from 640 to 650 led to a 1.51% increase, and from 740 to 750 a 0.46% increase. Increasing the diameter led to reaction force increases of 4.32% (640→740) and 3.25% (650→750). In tensile loading, increasing the length produced 1.71% (640→650) and 0.37% (740→750) force increases, while diameter increases produced 4.52% (640→740) and 3.15% (650→750) gains. Comprehensively analyzing these results, the increase in screw diameter can be inferred to enhance fixation strength, likely due to the expanded bone-screw contact area improving mechanical stability after implantation. Meanwhile, although theoretically a longer screw could contribute to higher fixation, experimental and simulation results in this study demonstrated that its structural impact was relatively minimal. Furthermore, excessively deep screw insertion could pose risks of unnecessary bone damage. Considering these aspects, a screw length of 40 mm, rather than 50 mm, appears to offer better efficiency and clinical safety. Additionally, as observed in the simulation results (Figure 8), increasing the screw diameter and length tended to shorten the yield displacement of the spinal rod. Based on these findings, the 640 case, with a 6 mm diameter and 40 mm length, exhibited the most balanced mechanical performance and clinical efficiency, as well as the greatest durability for maintaining spinal rod integrity. Therefore, the authors propose that the 640 configuration is the most biomechanically stable and efficient design for spinal fixation constructs under the tested conditions.

This study focused on the quantitative evaluation of spinal fixation construct mechanics based on the ASTM F1717 standard and assessed the feasibility of FEA-based structural validation. However, several limitations must be acknowledged.

First, this study evaluated construct-level behavior under static loading in the ASTM F1717 vertebrectomy configuration. Cyclic or fatigue behavior likely to occur *in vivo* was not addressed due to apparatus constraints and the predefined scope. Consequently, surrogate-material shear strength, fatigue strength, toughness, and hardness were not evaluated. In future work, we will incorporate high-cycle fatigue simulations based on the ASTM F1717 compression–bending framework, together with experimental validation, to more accurately reflect time-dependent reliability. We will also quantify shear strength, fatigue strength, toughness, and hardness.

Second, the material properties used in this study were not directly obtained through mechanical testing of the actual specimens. Instead, the FEA models adopted material parameters from validated literature and certified technical documents provided by manufacturers, a widely accepted practice in the field [V&V 40–2018, 2018; International Organization for Standardization, 2018; U.S. Food and Drug Administration, 2023]. Therefore, this study did not conduct reverse-engineering-based material testing for the specific specimens used. Instead, the modeling approach was grounded in external data sources whose reliability has been previously validated, thereby ensuring both realism and efficiency in the simulation framework.

Third, validation was conducted using reaction force at a single displacement point (20 mm) to compare experimental and simulation results quantitatively. While this approach effectively captured the mechanical response at a critical region, it may not fully represent the entire structural behavior. However, this study confirmed that yielding and permanent deformation of the spinal rod occurred prior to reaching a 20 mm displacement. This finding indicates that the structural system had already exceeded its mechanical threshold, thereby reaching a limit state directly related to patient safety. Accordingly, the deformation behavior beyond this point was considered to fall outside the clinically relevant and structurally meaningful range. Therefore, the comparative analysis between simulation and experiment was reasonably concluded at 20 mm displacement. In addition, stiffness, yield force, and yield displacement were used as ancillary comparison metrics to strengthen confidence in the experiment–simulation concordance.

Fourth, our experimental protocol cites F1717-15; however, the core procedural elements remain unchanged in F1717-21. We verified procedural equivalence by cross-checking loading rate and control mode, the measurement of active length, and the 2% offset yield definition. Therefore, the edition cited is not expected to materially affect the observed force–displacement metrics.

Fifth, we acknowledge that variability related to pilot-hole preparation and screw driving was not fully controlled in this study; aligning these steps with the relevant guidance in ASTM F543 may further reduce scatter. Consistent with our first limitation, future work will implement F543-consistent pilot-hole preparation and insertion-torque control, alongside high-cycle fatigue testing, to improve reproducibility.

In summary, this study is a foundational ASTM F1717-based parametric investigation that evaluates the effects of JIM/JEM, pedicle screw diameter, and pedicle screw length, and explores the feasibility of simulation-based validation as a substitute for selected physical tests. Future work will address the current limitations by incorporating fatigue analysis under clinically relevant conditions and time-dependent behavior. Additionally, future simulations will consider a broader range of parameters—such as screw pitch, insertion angle, and fixation location—to optimize the biomechanical efficiency and structural safety of spinal implants. Furthermore, high-resolution CT-based models will be utilized to develop patient-specific implant designs. The findings of this study are expected to assist clinicians in making more informed surgical decisions and ultimately contribute to improved outcomes in spinal fixation surgery.

## 5 Conclusion

This study demonstrated the feasibility and validity of using computational modeling and simulation (CM&S) as an alternative to physical testing for evaluating the mechanical performance of spinal fixation constructs in accordance with the ASTM F1717 standard. Static compression and tension tests were performed using both bench experiments and finite element analysis (FEA). The simulations agreed closely with the experimental data, especially for the reaction force at 20 mm displacement and stiffness. Mesh convergence and the simplification of boundary conditions ensured numerical accuracy and computational efficiency, enabling reliable and reproducible simulations.

## Data Availability

The original contributions presented in the study are included in the article, further inquiries can be directed to the corresponding author.
