# Supplementary material for: Simulation and validation of spinal construct testing based on ASTM F1717
Source: Front Bioeng Biotechnol. 2025 Nov 26;13:1673061. doi: 10.3389/fbioe.2025.1673061 (PMC12690298; doi:10.3389/fbioe.2025.1673061)
Supplement: Supplementary file 1 [file Supplementaryfile1.docx]

Supplementary Material

# Supplementary Figures and Tables

## Supplementary Figurs


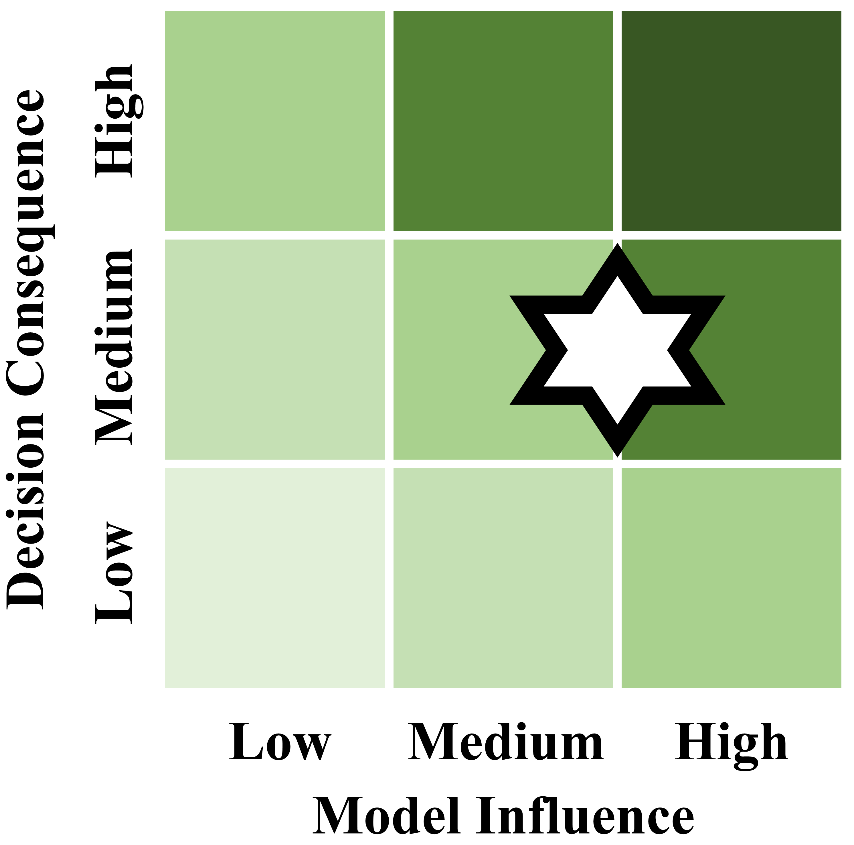


**Supplementary Figure 1.** Determining model risk

## Supplementary Table

**Supplementary Table 1.** Determining model risk

| **Evaluation factor** | **Definition (ASME V&V 40)** | **Application in this study** | **Level of assessment** |
| --- | --- | --- | --- |
| Model influence | (A) Simulation outputs from the computational model are a minor factor in the decision.  (B) Simulation outputs from the computational model are a moderate factor in the decision.  (C) Simulation outputs from the computational model are a significant factor in the decision. | Proposed to replace physical testing and inform design decisions | B~C (Medium~high) |
| Decision consequence | (A) An incorrect decision would not adversely affect patient safety or health, but might result in a nuisance to the physician or have other minor impacts.  (B) An incorrect decision could result in minor patient injury or the need for physician intervention, or have other moderate impacts.  (C) An incorrect decision could result in severe patient injury or death, or have other significant impacts. | Long-term reoperation risk under sustained load; not life threatening | B  (Medium) |
| Model risk | - | Model influence: Medium~high  Decision consequence: Medium | Medium~high |
| Validation level | Level 1 - Visual comparison concludes good agreement.  Level 2 - Comparison by measuring the difference between computational results and experimental data. Differences are less than 20%.  Level 3 - Comparison by measuring the difference between computational results and experimental data. Differences are less than 10%.  Level 4 - Comparison with uncertainty estimated and incorporated from the comparator or computational model. Differences between computational results and experimental data are less than 5%. Includes consideration of some uncertainty, but statistical distributions for uncertainty quantification are unknown.  Level 5 - Comparison with uncertainties estimated and incorporated from both the comparator and the computational model, including comparison error. Differences between computational results and experimental data are less than 5%. Statistical distributions for uncertainty quantifications are known. | Quantitative comparison (error ≤ 10%) with consideration of some uncertainties | Level 3~4 |
